# Late viral or bacterial respiratory infections in lung transplanted patients: impact on respiratory function

**DOI:** 10.1186/s12879-020-4877-3

**Published:** 2020-02-24

**Authors:** Marie Dubert, Benoit Visseaux, André Birgy, Pierre Mordant, Anne-Cécile Metivier, Gaelle Dauriat, Nadhira Fidouh, Yazdan Yazdanpanah, Nathalie Grall, Yves Castier, Hervé Mal, Gabriel Thabut, François-Xavier Lescure

**Affiliations:** 1 AP-HP, Hôpital Bichat, Service de maladies infectieuses et tropicales, 46 Rue Henri Huchard, F-75018 Paris, France; 2 INSERM, IAME, UMR 1137, F-75018 Paris, France; 3 Université Paris Diderot, IAME, UMR 1137, Sorbonne Paris Cité, F-75018 Paris, France; 4 AP-HP, Hôpital Bichat, Laboratoire de virologie, F-75018 Paris, France; 5 AP-HP, Hôpital Robert Debré, Laboratoire de microbiologie, F-75019 Paris, France; 6 AP-HP, Hôpital Bichat, Service de chirurgie thoracique, F-75018 Paris, France; 7 AP-HP, Hôpital Bichat, Service de pneumologie, F-75018 Paris, France; 8 AP-HP, Hôpital Bichat, Laboratoire de microbiologie, F-75018 Paris, France

**Keywords:** Viral respiratory infections, Lung graft patients, Bacterial respiratory infection, Lung graft acute rejection, Chronic lung allograft dysfunction

## Abstract

**Background:**

Respiratory infections are a major threat for lung recipients. We aimed to compare with a monocentric study the impact of late viral and bacterial respiratory infections on the graft function.

**Methods:**

Patients, who survived 6 months or more following lung transplantation that took place between 2009 and 2014, were classified into three groups: a viral infection group (VIG) (without any respiratory bacteria), a bacterial infection group (BIG) (with or without any respiratory viruses), and a control group (CG) (no documented infection). Chronic lung allograft dysfunction (CLAD) and acute rejection were analysed 6 months after the inclusion in the study.

**Results:**

Among 99 included lung recipients, 57 (58%) had at least one positive virological respiratory sample during the study period. Patients were classified as follows: 38 in the VIG, 25 in the BIG (among which 19 co-infections with a virus) and 36 in the CG. The BIG presented a higher initial deterioration in lung function (*p* = 0.05) than the VIG. But 6 months after the infection, only the VIG presented a median decrease of forced expiratory volume in 1 s; − 35 mL (IQR; − 340; + 80) in the VIG, + 140 mL (+ 60;+ 330) in the BIG and + 10 (− 84;+ 160) in the CG, *p* < 0.01. Acute rejection was more frequent in the VIG (*n* = 12 (32%)), than the BIG (*n* = 6 (24%)) and CG (*n* = 3 (8%)), *p* < 0.05, despite presenting no more CLAD (*p* = 0.21).

**Conclusions:**

Despite a less severe initial presentation, single viral respiratory infections seem to lead to a greater deterioration in lung function, and to more acute rejection, than bacterial infections.

## Background

Thanks to a better selection of recipients and donors, to an improvement in surgical / anaesthetic procedures and to better management of immunosuppressive therapies, early post-operative survival of lung transplant recipients (LTRs) has improved since the advent of lung transplantation.

Whereas most early deaths are related to primary graft dysfunction or acute rejection (AR), the long-term prognosis is threatened by chronic lung allograft dysfunction (CLAD), usually in the form of a bronchiolitis obliterans syndrome (BOS), that affects 45 to 75% of lung recipients within 5 years after transplantation [1–4]. CLAD represents the leading cause of death 1 year after lung transplantation [3, 5, 6]. Infectious respiratory complications are also a major cause of morbidity and mortality for LTRs and are responsible for a third of deaths occurring in the first year post transplant, and half of all deaths during long-term follow-up [7, 8]. The severity of these infections results from several factors including induced immunosuppression, direct exposure of the graft to microorganisms, and finally less effective mucociliary function and lymphatic drainage and cough reflex following denervation of the graft [9–11]. Moreover, stenosis and ischemic processes occurring at the surgical anastomosis decrease the clearance of secretions, and promote their colonization and invasion by microorganisms [12]. Bacterial infections are the leading cause of respiratory infections in LTRs [7, 11, 13]. Their association with the occurrence of CLAD is well established [2, 14].

Many authors admit that viral respiratory tract infections (VRTI) may be associated with CLAD [3, 15–24], but this remains controversial, depending on the definition of respiratory infection, the virus panel studied, the time limit between VRTI and spirometric analysis, and the consideration of intercurrent events possibly influencing the respiratory function [25]. Similarly, the association between VRTI and AR continues to be debated in the literature. While some studies have identified an association between these two events [17, 26, 27] some others, including a recent meta-analysis, have not found any link between them [21, 25, 28].

However, to our knowledge, the impact of viral-bacterial co-infections on graft survival has not been specifically studied, and was not compared to single VRTI or patients without any respiratory infections.

The development of rapid antigenic tests and molecular biology techniques has facilitated the detection and diagnosis of several viruses. The new multiplex PCR methods (Polymerase Chain Reaction) are fast, sensitive andable to detect an enlarged number of viruses not easily detected before (e.g. metapneumovirus, coronavirus NL63 and HKU1, bocavirus, rhinovirus C) [29, 30]. Thus, we hypothesis that the impact of the viral infection is at least as severe as bacterial respiratory infections on lung graft function among LTx. The objectives of our study were to assess, in a cohort study, the occurrence of late viral and bacterial respiratory infections in LTRs and to compare their respective impact on graft function with those without any respiratory infections.

## Methods

### Study design and patient recruitment

We retrospectively screened all individuals who underwent lung transplantation between September 2009 and September 2014 at the Bichat-Claude Bernard teaching hospital, Paris, France. In this cohort we evaluated the occurrence of late viral and bacterial respiratory infections and graft function overtime. Exclusion criteria were: (1) death during the first 6 months after transplantation, (2) no available pulmonary function assessment at the time of documented infection or 6 months after, and (3) herpetic or cytomegalovirus pneumonia.

In order to include patients at a steady state, we studied only the late respiratory infections, i.e. to censure the first 6 months after the lung transplantation, a period during which postoperative complications are frequent and usually intertwined. These patients were regularly followed up. At each routine follow-up visits with patients, symptoms were recorded. Spirometric measurements, blood tests, radiological explorations, bronchoscopic procedures with bronchoalveolar fluid and transbronchial biopsies were systematically performed and tested for both bacterial and viral pathogens according to local guidelines. All respiratory samples, bronchial aspirates, bronchoalveolar fluids and nasopharyngeal samples were reviewed.

Several mPCR tests were used during the study period: the Respifinder® 19 (Pathofinder®, Maastricht, the Netherlands) from September 2009 to February 2012, Respifinder® 22 (Pathofinder®, Maastricht, the Netherlands) from March to June 2012, and Filmarray Respiratory Panel RP1.6 (Biofire, BioMérieux, Craponne, France) was used from June 2012 to September 2014. All these changes were made to decrease time to results. The various tests used have been reported to have similar performances [31–33], as confirmed by our internal method validations and the similar viral diversity observed over time [34]. Their reliability was also assessed throughout the study period by regular QCMD controls (Glasgow, UK).

A sample was defined as bacteriologically positive if the bacteria were present at 10^7^ CFU/mL or more for sputum, 10^6^ CFU/mL or more for bronchial aspirates and 10^4^ CFU/mL or more for bronchoalveolar fluid.

According to virological and bacteriological results from respiratory samples, patients were divided into three groups, as follows:

- Patients presenting a viral respiratory infection: viral infection group (VIG), i.e.
- Patients with at least one positive virological respiratory sampling, symptomatic or not. Nasopharyngeal swabs that tested positive only for rhinovirus were not considered to be positive for a significant virus, and were ignored.
- Patients presenting a bacterial respiratory infection with or without any respiratory virus: bacterial infection group (BIG), i.e. (i) patients with a positive bacteriological sample treated with antibiotics, with or without a positive virological sample during the study period (ii) patients with no bacteriological positive sample but treated with antibiotic therapy before the taking of the samples.
- Patients without any respiratory infection: Control group (CG), i.e. patients with no virological or bacteriological positive respiratory samples during the study, or those with nasopharyngeal swabs positive for rhinovirus only; or with a positive bacteriological respiratory sample not followed by antibiotic treatment (i.e. considered as a simple bacterial carriage).

The date of inclusion was defined as the date of the respiratory sample of interest for the two infected groups (VIG and BIG), and the date of the first outpatient consultation for the CG.

### Outcomes

The primary endpoint was the occurrence of BOS 6 months after inclusion. Secondary endpoints were the occurrence of AR and the quantitative change of forced expiratory volume in 1 s (FEV-1) at 6 months after inclusion, as well as death during the whole study period.

The following definitions were used to assess the outcomes:

1. BOS was defined according to the International Society for Heart & Lung Transplantation (ISHLT) algorithm based on forced expiratory volume in 1 s (FEV-1) values [8].
2. BOS-worsening was defined as a worsening in the previously defined stage of BOS.
3. AR was defined on the basis of a transbronchial biopsy that demonstrated at least a Grade A1 of acute rejection as defined by the ISHLT for cellular rejection. In patients in whom a biopsy could not be performed, acute rejection was defined by deterioration in lung function with no other identifiable aetiology and that positively responded to a high-dose corticosteroid therapy.
4. FEV-1 delta was defined as 6-month-FEV-1 – last FEV-1 before inclusion.

### Statistical analysis

Quantitative variables were presented using the median (quartile 25–75) and were compared by t-test or variance analysis. Qualitative variables were compared using a Chi2 test or a Fisher-exact test. Outcomes were compared using a logistic regression multivariable model, with adjustment for time since transplantation, age and sex. A sensitivity analysis was carried out excluding patients who had had a BOS before inclusion. All of the statistical analysis was done using R software, version (3.1.1).

### Ethics approval

This study was approved by the CEPRO (Comité d’Evaluation des Protocoles de Recherche Observationnelle) ethical committee, number CEPRO 2016–027.

## Results

### Patients and baseline characteristics

**Fig. 1:**
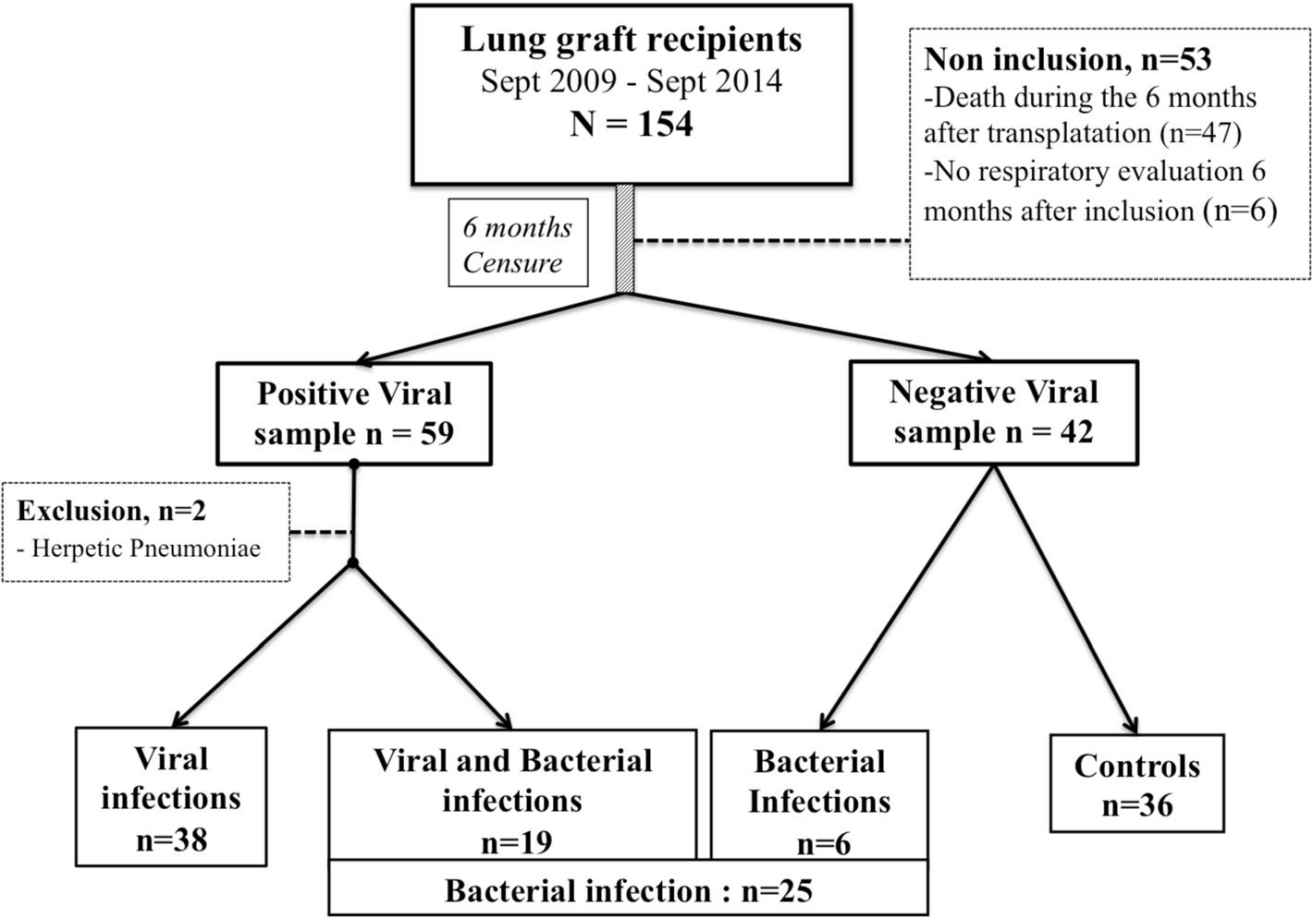
Flow chart. After the exclusion of 53 patients, 99 lung transplant recipients were included. Among them, 59 presented at least one positive respiratory virologic sample during the follow-up. Patients were divided into 3 groups viral infection (*n* = 38), bacterial infection (*n* = 25) and controls (*n* = 36)

Between September 2009 and September 2014, 154 patients received lung transplants at Bichat Hospital. Fifty-five patients were excluded from the analysis: 47 (31%) because of death within 6 months following transplantation, six (4%) due to a lack of spirometry assessment at inclusion or 6 months afterwards, and two because of pathological, proven herpetic pneumonia. Among the 99 remaining LTRs, 57 (57%) had at least one positive virological respiratory sample. Thus, patients were divided into three groups according to the criteria described above: 38 (38%) in the VIG, 25 (25%) in the BIG (6 patients mono-infected by bacteria and 19 co-infected patients) and 36 (36%) in the CG. The corresponding flow chart is presented in Fig. 1. Nineteen 4 % of the respiratory samples were broncho-alveolar lavages or bronchial aspirations. General characteristics, type of immunosuppression and prior viral infections were similar across the three groups with the exception of renal dysfunction (eGFR < 60 mL/min/1.73m^3^), more prevalent in the VIG (Table 1). Previous AR requiring treatment in the 3 months prior to inclusion occurred for 9 (24%) patients in the VIG, 3 (12%) in the BIG, and none of the CG (*p* < 0.01). Among 13 patients presenting a BOS at inclusion, 9 were classified in VIG, compared to 3 in the BIG, and 1 in the CG (*p* = 0.02).

**Table 1:** Clinical and spirometric characteristics at baseline. Description and comparison between the three groups

	Total	VIG	BIG	CG	*p*-value
	*N* = 99	N = 38	N = 25	N = 36	
Main characteristics					
Age in years, median (IQR)	57.5 (48.6–62.0)	57.7 (52.4–60.3)	57.7 (46.2–62.0)	56.3 (49.9–63.3)	0.92
Masculine gender, n (%)	64 (65)	27 (71)	18 (72)	19 (53)	0.17
Comorbidities at enrollment					
Renal dysfunction, n (%)	9 (9)	8 (21)	1 (4)	0 (0)	0.01
Diabetes mellitus, n (%)	63 (64)	23 (61)	20 (80)	20 (56)	0.13
Arterial hypertension, n (%)	53 (54)	22 (58)	14 (56)	17 (47)	0.63
Underlying respiratory diseases					
Bronchial dilatation (%)	6 (6)	2 (5)	3 (12)	1 (3)	0.38
Emphysema/COPD (%)	30 (30)	14 (37)	8 (32)	8 (22)	0.38
Alpha-1 antitrypsine deficiency (%)	8 (8)	2 (5)	2 (8)	4 (11)	0.74
Pulmonary fibrosis (%)	42 (42)	15 (40)	10 (40)	17 (47)	0.77
Other interstitial disease (%)	13 (13)	5 (13)	2 (8)	6 (17)	0.65
Immunosuppresive therapy					
Tacrolimus (%)	89 (90)	35 (92)	24 (96)	30 (83)	0.27
Mycophenolate mofetil (%)	83 (84)	31 (82)	24 (96)	29 (81)	0.41
Graft features					
Second graft (%)	3 (3)	2 (5)	1 (4)	0 (0)	0.47
Bilateral lung transplant (%)	26 (26)	9 (24)	8 (32)	9 (25)	0.75
Prior viral respiratory infection (%)	6 (6)	2 (5)	3 (12)	1 (3)	0.38
Treatment for AR 3 months prior to inclusion (%)	12 (12)	9 (23)	3 (12)	0 (0)	< 0.01
Spirometric characteristics					
Last FEV-1 (mL/s)	1670 (1410–2100)	1670 (1410–2210)	1620 (1410–2100)	1770 (1498–2062)	0.49
BOS prior to enrollment^a^ (all stage) (%)	13 (13)	9 (24)	3 (12)	1 (3)	0.02

*Abbreviations: AR* acute rejection; *BIG* bacterial infections group; *CG* control group; *COPD* Chronic obstructive pulmonary disease; *FEV-1* forced expiratory volume in 1 s; *VIG* viral infections group

^*a*^ *last FEV-1/best FEV-1*

### Clinical presentation (Table 2)

Patients were included at a median time of 303 (IQR, 213–560) days after transplantation. Both infected groups displayed similar respiratory symptoms. Likewise, the biological presentations of both infected groups were close, and inflammatory surrogates (leucocytes, C-reactive protein, platelets) did not differ statistically. BIG patients were more prone to be hospitalized (64 vs. 34%, *p* = 0.04), and to present a more severe decrease of FEV-1 at inclusion (− 260 mL (− 410–0) vs. -50 mL (− 170–60)), compared to the VIG. Among patients examined with a thoracic tomodensitometry, seven patients (11%) presented a new infiltrate.

**Table 2:** Characteristics of the infection disease at baseline. Comparison between the two infected groups

	TOTAL	VIG	BIG	*p*-value^b^	CG
	*N* = 63	*N* = 38	*N* = 25		*N* = 36
Time from transplant in days, median (IQR)	303 (213–560)	366 (250–615)	255 (198–460)	0.27	204 (190–232)
Hospitalization, n (%)	29 (46)	13 (34)	16 (64)	0.04	3 (8)
Time of hospital stay in days, n (%)	14 (8–22)	11 (7–15)	16 (12–23)	0.11	25 (16–26)
Symptoms	41 (73)	23 (70)	18 (78)	0.69	2 (6)
Temperature, °C, (median, IQR)	36.8 (36.4–37.0)	37.0 (36.6–37.0)	36.7 (36.4–36.8)	0.28	36.7 (36.5–36.9)
Desaturation < 95% AA (n, %)	11 (25)	3 (13)	8 (40)	0.08	0 (0)
Dyspnea (n, %)	21 (43)	9 (33)	12 (55)	0.23	1 (3)
Cough (n, %)	28 (54)	16 (52)	12 (57)	0.91	1 (3)
Sputum (n, %)	23 (43)	10 (32)	13 (59)	0.10	0 (0)
Coryza (n, %)	12 (23)	7 (21)	5 (25)	0.75	1 (3)
Auscultator abnormality (n, %)	14 (28)	8 (27)	6 (30)	1.00	0 (0)
Biology					
Leucocytes, G/L (median, IQR)	8190 (5180–1044)	8100 (5950–11,070)	8205 (4695–16,450)	0.49	8280 (6215–9910)
Hemoglobin, g/dL (median, IQR)	11.6 (10.7–12.3)	11.5 (10.7–12.3)	11.6 (10.1–12.4)	0.97	11.4 (10.4–12.3)
Platelets, 10^3^ G/L (median, IQR)	250 (216–303)	248 (216–321)	270 (218–302)	0.69	261 (202–329)
C-Reactive Protein, mg/L (median, IQR)	6.5 (5.0–34.8)	5.5 (5.0–26.3)	8.5 (5.0–55.5)	0.51	5.0 (5.0–9.0)
Creatininemia, μmol/L (median, IQR)	111 (85–139)	114 (85–143)	105 (86–126)	0.18	89 (76–105)
GFR, ml/min/1.73 (median, IQR)	55 (44–78)	56 (41–78)	52 (47–74)	0.93	72 (57–81)
Tacrolimus level, UI/L (median, IQR)	8.8 (7.3–10.8)	8.6 (7.1–10.5)	9.4 (8.0–10.9)	0.91	9.2 (7.7–10.2)
New infiltrate in thoracic TDM (n, %)	7 (11)	3 (9)	4 (20)	0.37	3 (10)
Delta FEV-1 at inclusion^a^, ml (median, IQR)	−45 (− 188–68)	−50 (−170–60)	−260 (−410–0)	0.05	0 (−95–74)
Antiviral therapy (n, %)	21 (33)	14 (37)	7 (28)	0.65	0 (0)
Antibiotic therapy (n, %)	28 (44)	3 (8)	25 (100)	< 0.01	0 (0)

*Abbreviations: BIG* bacterial infections group; *CG* control group; *GFR* Glomerular filtration rate; *TDM* tomodensitometry; *FEV-1* forced expiratory volume in 1 s; *VIG* viral infections group

^a^ *Inclusion FEV-1 - last FEV-1*

^b^ Comparison *between the two infected groups, viral and bacterial infections*

### Microbiology findings

Picornavirus was the most frequently detected virus in the VIG (*n* = 21), followed by parainfluenzae virus (*n* = 13), and syncytial respiratory virus (*n* = 10) (Table 3). *Pseudomonas aeruginosa* was the main detected bacteria; found in 11 (52%) patients in the BIG. In the CG, the neglected bacteria were *Pseudomonas aeruginosa* (*n* = 4), *Corynebacteriae striatum* (n = 2) and others (n = 2). Three patients had more than one detected virus and eight patients had more than one detected bacteria.

**Table 3:** Description of microbiological findings in the Viral Infection Group (VIG) and Bacterial Infection Group (BIG)

	Total	VIG	BIG
	*N* = 63	*N* = 38	*N* = 25^a^
Number of samples during the study period, median per patient (IQR)	12.0 (8.0–17.5)	12.0 (8.0–17.8)	12.0 (10.0–17.0)
Virological aetiology			
Picornaviruses, n (%)	21	10 (24)	11 (52)
Parainfluenzae viruses, n (%)	13	11 (29)	2 (8)
Respiratory syncytial viruses, n (%)	10	6 (16)	4 (16)
Coronaviruses, n (%)	8	6 (16)	2 (8)
Human metapneumovirus, n (%)	5	4 (11)	1 (4)
Influenza viruses, n (%)	3	2 (5)	1 (4)
Adenovirus, n (%)	1	0 (0)	1 (4)
Bacteriological aetiology			
*Pseudomonas aeruginosa*, n (%)	15	4 (11)	11 (52)
*Corynebacteriae striatum*, n (%)	7	1 (3)	7 (24)
*Enterobacteriaceae*, n (%)	5	2 (5)	3 (12)
*Staphylocoque*, n (%)	2	1 (3)	1 (4)
*Streptocoque*, n (%)	1	0 (0)	1 (4)
Other bacteria, n (%)	2	2 (5)	0 (0)

*Abbreviations: BIG* bacterial infections group; *VIG* viral infections group

^a^ *Among 25 patients with bacterial infections, 7 had no bacteria identification, but samples were examined after antibiotic therapy (n = 3) and/or presented a localized chest X-ray condensation (n = 3) and/or patients had purulent sputum (n = 2)*

### Outcomes

The overall rate of worsening 6-month-BOS was 13% with no significant differences between the three groups (Table 4). Sixteen patients (16%) died during the study period (6 BOS, 2 strokes, 2 neoplasia, 2 severe infections, 4 from unknown cause), with no significant differences between the three groups (Table 4). After 6 months of observation, the FEV-1 delta was significantly different between the three groups, from a negative delta at -35 mL (IQR: − 340; + 80) in the VIG, to a median increase of + 10 mL (− 85; + 160) in the CG, and + 140 mL (+ 60; + 330) in the BIG (*p* < 0.01). The rate of AR was significantly different between the three groups: 32% of the VIG, 24% of the BIG and 8% of the CG (overall *p* < 0.05). AR occurred earlier in the BIG, at a median of 6.5 (2.3–32.5) days, than in the VIG at 79.5 (5.0–111.2) days (*p* = 0.03). In multivariate analysis there was a trend toward a higher risk of 6-month-BOS in the VIG vs. BIG (adjusted OR = 6.4 [0.84 to 48.8], *p* = 0.07). In multivariate analyses adjusted for time between transplant and inclusion, age and sex, the association between AR and VIG remained significant although with a wide confidence interval (aOR = 5.5 [1.3–24.0], *p* = 0.02). (Table 5) In the two infected groups, these outcomes were not different between the symptomatic patients (*n* = 41) and the asymptomatic patients (*n* = 15).

**Table 4:** Outcomes (Delta-FEV-1, Acute rejection, BOS and Death) after 6 months of observation. Description and univariate comparison between the viral infection group (VIG), the bacterial infection group (BIG) and the control group (CG)

	Total	VIG	BIG	CG	*p*-value
	*N* = 99	*N* = 38	*N* = 25	*N* = 36	
Delta FEV-1 (mL), median, (IQR)	40 (−95; + 160)	−35 (−340; + 80)	+ 140 (+ 60;+ 330)	+ 10 (−84;+ 160)	< 0.01
AR, n (%)	31 (31)	12 (32)	6 (24)	3 (8)	< 0.05
BOS, n (%)	13 (13)	8 (21)	2 (8)	3 (8)	0.21
Death, n (%)	16 (16)	10 (26)	3 (12)	3 (8)	0.10

*Abbreviations: AR* Acute rejection; *BIG* Bacterial infection group; *BOS* bronchiolitis obliterans syndrome; *CG* control group; *FEV-1* forced expiratory volume in 1 s; *VIG* viral infection group

**Table 5:** Outcomes (BOS and AR) after 6 months of observation. Description and multivariable comparison between the viral infection group (VIG), the bacterial infection group (BIG) and the control group (CG)

	N (%)	Adjusted OR	95% CI	*p*-value^a^
6-month-BOS				
CG (*n* = 36)	3 (9%)	1	1	1
VIG (*n* = 38)	8 (23%)	3.0	[0.66–14.0]	0.16
BIG (*n* = 25)	2 (8%)	0.8	[0.11–5.8]	0.83
6-month-AR				
CG (*n* = 36)	3 (8%)	1	1	1
VIG (*n* = 38)	12 (32%)	5.5	[1.25–24.0]	0.02
BIG (*n* = 25)	6 (24%)	3.7	[0.77–18.0]	0.10

*Abbreviations: AR* Acute rejection; *BIC* Bacterial infection group; *BOS* bronchiolitis obliterans syndrome; *CG* control group; *CI* Confidence Interval; *OR* odds ratio; *VIC* viral infection group

^*a*^ *with adjustment for time between transplant and inclusion, age and sex*

### Sensitivity analysis

Thirteen patients had a prior BOS at inclusion: 1 (2.8%) in the CG, 9 (23.7%) in the VIG and 3 (12.0%) in the BIG. There was a trend toward more viral respiratory investigation among patients with a BOS at inclusion (median of 14.0 (9.0–21.0) samples/patients) than for patients without BOS at inclusion (8.0 (4.0–15.0), *p* = 0.06). However, the sensitivity analysis performed on the remaining 86 patients without BOS at inclusion found similar results in terms of significance and association effect with the development of a new BOS or with AR (Table S1). In the same way, infected patients were significantly more investigated than those in the CG: respectively 12.0 (8.0–17.8), 12.0 (10.0–17.0) and 4.0 (3.0–7.3) samples per patient for the VIG, BIG and CG (*p* < 0.001).

## Discussion

This study shows that, after the first 6 months following transplantation, more than half of LTRs were affected by VRTI. Clinical presentations for late viral and/or bacterial infections at baseline were very similar, albeit with additional signs of severity for the bacterial infections. Single late VRTI strongly impacted the patients’ prognosis by leading to an increased risk of AR, a trend to an increased risk of BOS (without significant association), and a more severe secondary decline in respiratory function compared to the late bacterial respiratory infection. The consequences of these different infections were similar whether or not the infection was symptomatic at the time of viral or bacterial detection.

In this study, 57 patients (57%) exhibited at least one positive viral respiratory sampling during follow-up. This rate varies among studies as do screening techniques and reasons for withdrawal. While studies using cell cultures in their screening method report virus detection rates in respiratory samples at around 8% [2, 15], the use of molecular biology tests significantly increases this prevalence from 17 to 52% [17, 28, 35]. We decided to exclude infections with CMV, especially because CMV diseases respond to different triggers than those infections, were prevented by different protocol evolutions during the study period, and can induce the death or graft rejection in both of our patient groups. With regard to microbiological aetiology, we confirmed that the most frequent viruses detected with the PCR test were those corresponding to the picornavirus group, followed by parainfluenzae viruses and coronaviruses, as already described [5, 6, 18, 23, 24, 27]. It is worthy of note that, in the BIG, picornavirus were the most frequently detected viruses in co-infections (52%). Picornavirus are often considered as a contaminant with a controversial clinical impact. Indeed, a recent prospective study demonstrated that rhinoviruses were frequent in LTRs, even in those patients who were asymptomatic [35]. Influenza were rarely identified among lung graft patients, especially when compared to the non-lung graft patients in our hospital using the same mPCR assays (5 vs 27%) [34]. This is explained by the high vaccination rates and specific prevention measures compliance among lung graft patients and their relatives. Among bacterial infections, *Pseudomonas aeruginosa* and *Corynebacteriae striatum* were more often detected. Both bacteria are known to be responsible for serious infections in LTRs. As already shown, the symptomatic feature of the initial infection did not impact on graft survival [15, 17, 25], suggesting that, for these patients, the symptomatic nature of the infection should not be taken into account. Concerning AR, the impact of late respiratory viral and/or bacterial infections on the graft function was significantly different with three times more AR within 6 months for both the VIG and BIG compared to the CG. While some studies supported this association [16, 36], other studies, including a meta-analysis, did not find any significant link [17, 25, 28, 30]. This difference could be explained by the variety of criteria used to define AR. We chose to identify AR when the histological pattern showed a stage of at least A1. Indeed, previous studies demonstrated that minimal rejection (≥A1) was associated with an increased risk for BOS development and progression that was comparable to A2 rejection [37]. On top on that, we noticed a significantly longer delay in AR in the VIG than in the BIG, suggesting that the impact of viral infection on lung graft function must be screen even after several weeks.

Especially in asymptomatic LTRs and the lack of specific management, the morbidity of viral infection could be attributed to a trivialization of viral colonization, leading to a neglected and chronic cause of inflammation and, thus, to potential rejection. Therefore, it seems important to assess the impact of respiratory viral infections on the graft function: to emphasize the prevention of viral infections for immunocompromised with more frequent sampling of patients including wide respiratory virus detection by molecular techniques and to strengthen spirometric controls after viral infections. The all-cause mortality was evaluated to 31% at 6 months in this study. This rate in consistent with other studies [8, 15]. It is explained by the high rate of comorbidity among our patients (more than half with arterial hypertension and diabetes mellitus), the deep immunosuppression required and the numerous complications of lung grafting.

To our knowledge, this study is the first to allow a direct comparison of the impact of late viral and bacterial respiratory infections in LTRs. We were able to analyze all the spirometry data both at inclusion and 6 months after, and to present results of an extensive panel of viral PCR tests.

This study has several limitations. Firstly, the small number of patients could restrict the power of the conclusions, especially for patients classified only on nasopharyngeal swabs (*n* = 4). aORs present wide ranges and must be interpreted accordingly. However, this study remains one of the largest available cohort on the topic to date. Despite this small sample size we were able to illustrate that late VRTI strongly impacted the patients’ prognosis by leading to an increased risk of AR. In addition, although not significant there was a trend to higher risk of occurrence of other outcomes such as BOS and death with VRTI. We believe that these findings are important and should lead to the design of larger studies. Because of the small number of patients among the group with bacterial infections and the group with bacterial and viral infections, we decided to merge these two groups and this could be also debated. However, this was done first because of the similar presentation of patients in these groups and second on the basis of the hypothesis that: (i) bacterial infections were responsible of the major acute part of the lung graft malfunction, and (ii) these groups were both subject to an intervention with the use of antibiotics. Secondly, the retrospective design leads to several biases: an indication bias leading to higher infection detection in patients presenting AR 3 months prior to inclusion or a BOS prior to infection because of more frequent follow-up visits and a trend to more respiratory sampling for these patients. Although treatment for AR 3 months prior to inclusion could be considered as a biais, this rate was not significantly different between the infected groups. Because of the small number of patients, we could not perform a sensitivity analysis among these patients who had AR 3 months prior to inclusion.

Nevertheless, we performed a sensitivity analysis to address the hypothesis of indication bias. We repeated the multivariable model without considering patients with a BOS at inclusion and found similar results, suggesting that this bias, if it existed, did not alter our conclusions. We also assume a survival bias that may explain why despite higher lung function deterioration at 6 months after inclusion in the viral infections group, the rate of BOS was not significantly different. The relatively short 6 months follow-up period used in our study may not be sufficient to identify a potential difference in the progression of BOS between groups. Indeed, the incidence of BOS development 6 months after the first infection episode was at 13% in our study, while previous studies described an incidence rate of 50% over a five-year period [2].

## Conclusions

To conclude, viral respiratory infections and virus-bacteria co-infections were frequent in lung graft recipients and led to similar clinical and biological presentations. Bacterial infections strongly diminished initial lung function and led to more hospitalizations whereas single viral respiratory infections seem to lead to a greater deterioration in lung function, and to more acute rejection, than bacterial infections.

## Supplementary information

**Additional file 1: Table S1.** Multivariate analysis of association with development of BOS. Without patients with BOS at inclusion

## Data Availability

The datasets used and/or analysed during the current study are available from the corresponding author on reasonable request.

## Abbreviations

AR: Acute rejection
BIG: Bacterial infection group
BOS: Bronchiolitis obliterans syndrome
CG: Control group
CLAD: Chronic lung allograft dysfunction
FEV-1: Forced expiratory volume in 1 s
ISHLT: International Society for Heart & Lung Transplantation
LTRs: Lung transplant recipients
PCR: Polymerase Chain Reaction
VIG: Viral infection group (VIG)
VRTO: Viral respiratory tract infections
