# Supplementary material for: Late viral or bacterial respiratory infections in lung transplanted patients: impact on respiratory function
Source: BMC Infect Dis. 2020 Feb 24;20:176. doi: 10.1186/s12879-020-4877-3 (PMC7041086; doi:10.1186/s12879-020-4877-3)
Supplement: Supplementary file 1 — Additional file 1: Table S1. Multivariate analysis of association with development of BOS. Without patients with BOS at inclusion [file 12879_2020_4877_MOESM1_ESM.docx]

**Supplemental data**

**Different mPCR tests used during the study period**

During the study period, different mPCR tests were used: the Respifinder® 19 (Pathofinder®, Maastricht, Netherlands) ^20^ from May 2011 to February 2012, Respifinder® 22 (Pathofinder®, Maastricht, Netherlands) ^21^ from March 2012 to December 2013, and Anyplex^TM^ II RV16 (Seegene®, Seoul, South Korea) ^22^ from January to April 2014. From June 2012 to April 2014, the Filmarray Respiratory Panel (BioFire Diagnostics, Salt Lake City, USA) ^23^ was also used when a fast-track result was specifically requested by the physicians. All these tests allowed the detection of rhinovirus, enterovirus, human metapneumovirus, coronavirus (NL63, HKU1, 229E, OC43), respiratory syncytial virus (A/B), influenza (A/B), parainfluenza virus (1 to 4) and adenovirus. Only Respifinder^®^ 22 and Anyplex^TM^ II RV16 were able to detect bocavirus and to differentiate rhinovirus and enterovirus. Thus, rhinovirus and enterovirus results were grouped as picornavirus for the analysis of the results depicted in this study. All these techniques were compared in our laboratory and the literature, showing comparable performances ^21,22,24^ and were validated locally with the use of a European quality control standard (QCMD, Glasgow, UK).

**Table S1: multivariate analysis of association with development of BOS. Without patients with BOS at inclusion**

|  | **N (%)** | **Adjusted OR** | **[95% CI]** | **p-value** |
| --- | --- | --- | --- | --- |
| **6-months BOS** |  |  |  |  |
| Controls (n=35) | 3 (10%) | 1 | 1 | 1 |
| Viral infections (n=29) | 7 (24%) | 3.5 | [0.72-17.4] | 0.12 |
| Bacterial infections (n=22) | 2 (10%) | 0.6 | [0.07-5.0] | 0.63 |
| **Acute Rejection** |  |  |  |  |
| Controls (n=35) | 3 (9%) | 1 | 1 | 1 |
| Viral infections (n=29) | 8 (28%) | 5.4 | [1.16-25.3] | 0.03 |
| Bacterial infections (n=22) | 5 (23%) | 3.8 | [0.74-20.0] | 0.11 |

*Abbreviations: BOS, Bronchiolitis obliterans syndrome; CI, Confidence Interval; OR, odds ratio*
